# Morphological and molecular dissection of wild rices from eastern India suggests distinct speciation between *O. rufipogon* and *O. nivara* populations

**DOI:** 10.1038/s41598-018-20693-7

**Published:** 2018-02-09

**Authors:** Rashmita Samal, Pritesh Sundar Roy, Auromira Sahoo, Meera Kumari Kar, Bhaskar Chandra Patra, Bishnu Charan Marndi, Jwala Narasimha Rao Gundimeda

**Affiliations:** Division of Crop Improvement, National Research Institute, Cuttack, 753006 India

## Abstract

The inter relationships between the two progenitors is interesting as both wild relatives are known to be the great untapped gene reservoirs. The debate continues on granting a separate species status to *Oryza nivara*. The present study was conducted on populations of *Oryza rufipogon* and *Oryza nivara* from Eastern India employing morphological and molecular characteristics. The cluster analysis of the data on morphological traits could clearly classify the two wild forms into two separate discrete groups without any overlaps i.e. lack of intermediate forms, suggesting the non-sympatric existence of the wild forms. Amplification of hyper variable regions of the genome could reveal 144 alleles suggesting high genetic diversity values (average He = 0.566). Moreover, with 42.37% of uncommon alleles between the two wild relatives, the molecular variance analysis (AMOVA) could detect only 21% of total variation (p < 0.001) among them and rest 59% was within them. The population structure analysis clearly classified these two wild populations into two distinct sub-populations (K = 2) without any overlaps i.e. lack of intermediate forms, suggesting the non-sympatric existence of the wild forms. Clear differentiation into two distinct groups indicates that *O. rufipogon* and *O. nivara* could be treated as two different species.

## Introduction

Rice, the world’s most important food crop and primary food source for more than one third of the world’s population, accounts for around half of the calories consumed by three billion Asians. To meet the future demands of food needs of the population, there is an urgent need to enhance the productivity of rice that has reached a plateau due to a narrow genetic base of the modern ricecultivars^1^. The crop improvement programs are dependent on continuous infusions of genes/QTLs from landraces and wild relatives to address the genetic enhancement programs and this path requires an assessment of diversity in the base populations to select desirable genotypes for use. Though utilization of natural variation available in the landraces is a continuous ongoing process of the crop improvement programs, it is important to explore the wild germplasm forms with novel traits as wild relatives of cultivated crop species are increasingly recognized as a valuable repository of useful variation. Wild species of rice are reservoirs of many useful genes but a vast majority of these genes remained untapped.

*Oryza rufipogon* and *Oryza nivara*, the two closest relatives of Asian cultivated rice (*O. sativa* L.) are considered to be its progenitors^2,3^. As the wild relatives of rice can be an important source of beneficial genes in rice breeding, *O. rufipogon* and *O. nivara* have long been the subject of extensive taxonomic, phylogenetic and population studies^3,4^.

*O. rufipogon* is a perennial, photoperiod sensitive, largely cross-pollinated, grows in habitats that include swamps and lakes, areas with year round assured water availability while *O. nivara* is an annual, photoperiod insensitive and predominantly self-pollinated species and adapted to dry habitats that lack of ponded water. While the distribution of *O. rufipogon* is over a wide area comprising South and South east Asia, Papua New Guinea and Australia, *O. nivara* is restricted to South and Southeast Asia^5,6^.

*Oryza nivara* is considered as a distinct species^7–10^ or as an ecotype of *O. rufipogon*^2,4,11^ and its taxonomic status is not clear due to the inconsistent reports. The data derived from isozymes^12^, RAPIDs^13^, allozymes and RFLPs^14^, transposon display markers^15^, tourist sequences^16^, miniature inverted-repeat transposable elements inAFLPs^17^, SSRs^13^, STSs^18^ and various gene sequences^19–21^ did not detect any significant divergence between *O. nivara* and *O. rufipogon*, while the data derived from AFLPs^22^, microsatellites^23,24^, combined sequence data from chloroplast, mitochondrial and nuclear DNA^25^ and SNPs^26^ did indicate differences to grant a species status to *O. nivara*.

Till date, the contribution of two wild progenitors to rice improvement is significant. The hybrid rice era in rice has started with the discovery of the WA (wild abortive) cytoplasm in *O. rufipogon*^27^ and some of the other important beneficial traits from *O. rufipogon* include yield QTLs, resistance to rice tungro virus and elongation ability are of great importance for rice breeding^28,29^ while the widely used resistance to grassy stunt virus in cultivated rice is unique to *O. nivara* and not observed in any other species or cultivated rice germplasm.

However, populations of these species are rapidly declining due to changes in farming systems, economic development, urbanization, industrial exploitation and other human disturbances in the past few decades.The understanding of the extent and type of genetic variation of wild rice populations can help in identification of valuable accessions and conservation and utilization strategies can be formulated. Though morphological data is known to be of great value for assessment and comparison of diversity patterns within and among populations^30^, the advent and advances in molecular techniques has provided us opportunities to assess the variation at molecular level. The molecular techniques are well known for their use in analysis of specific genes, understanding gene action, genetic map generation, phylogenetic studies and species evolution that can also offer help in understanding the extent and distribution of genetic variation within and between species.

The present study reports the results based on detailed investigations on morphological characteristics and several loci at molecular level in *O. nivara* and *O. rufipogon* populations with an objective to understand their inter relationship and evolutionary aspects.

## Results

### Morphological traits

**Figure 1:**
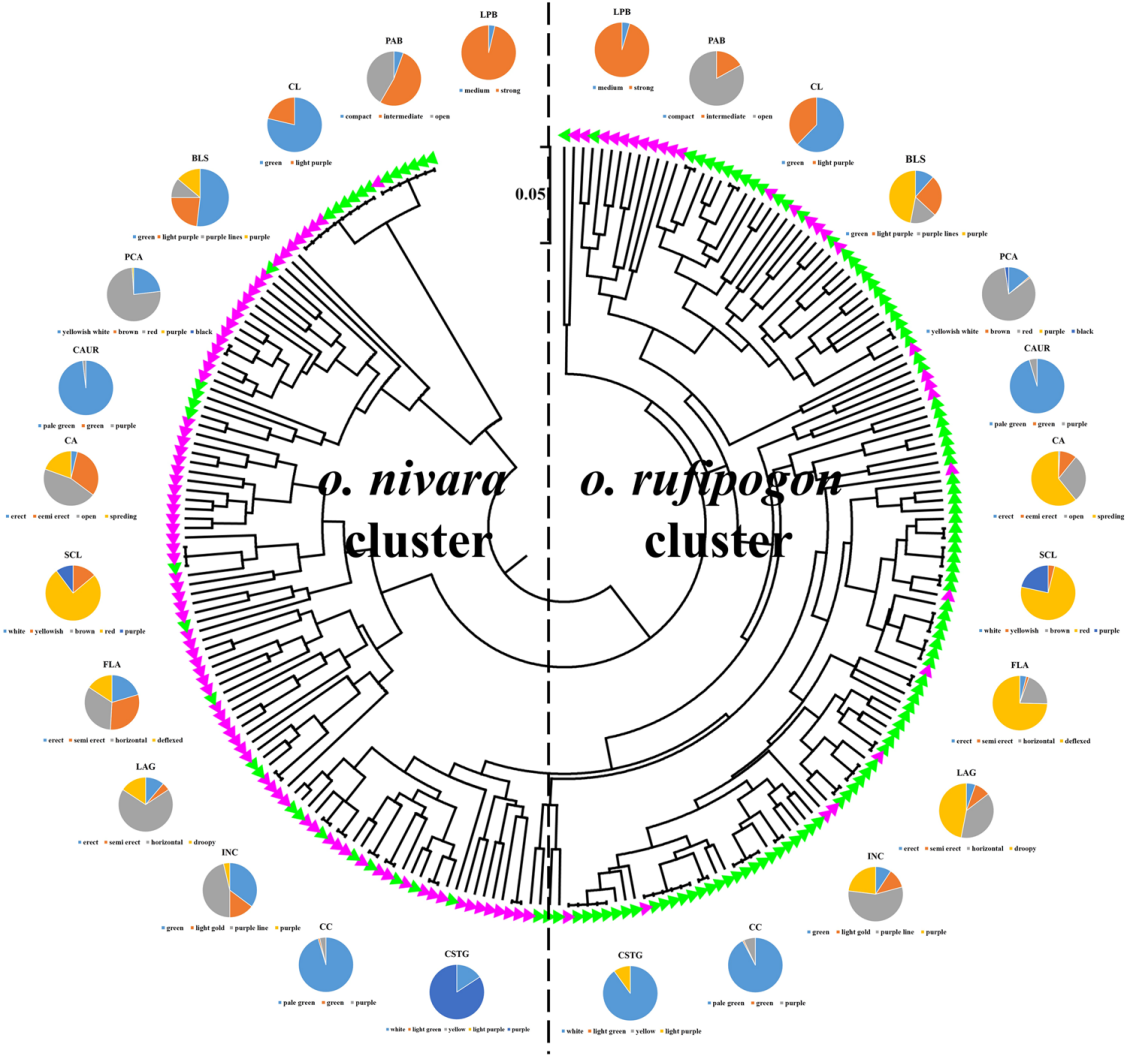
Qualitative morphological trait diversity among *O. rufipogon* and *O. nivara* accessions and differentiation of both the groups based on neighbor-joining dendrogram with qualitative morphological description. *Note: Please refer Supplementary Table* S1 *for description of each figure legends*.

*O. nivara* and *O. rufipogon* populations, both showed high level of variation for their qualitative morphological traits. Of the 22 characters, 19 and 17 showed variation in the *O. nivara* and *O. rufipogon* populations, respectively (Supplementary Table S1). In the *O. nivara* population no variation was detected for panicle: awns (present in all accessions), leaf: shape of ligule (split) and leaf: auricles (present), all being monomorphic. Similarly, the characters like panicle: awns (present), panicle: curvature of main axis (straight), panicle: secondary branching (weak), leaf: shape of ligule (split) and leaf: auricles (present) could not identify any difference among the *O. rufipogon* accessions. Among the *O. nivara* accessions, highest variation was detected for basal leaf: sheath colour [four alternative forms i.e. green (52%), light purple (23%), purple line (11%), purple (14%)], leaf: angle [erect (11%), semi erect (5%), horizontal (69%), droopy (16%)], culm: attitude [erect (4%), semi erect (31%), open (45%), spreading (19%)], internodes: colour [green (35%), light gold (15%), purple line (46%), purple (4%)] and flag leaf: attitude of blade [erect (20%), semi erect (31%), horizontal (33%), deflexed (16%)](Fig. 1). Further, basal leaf: sheath colour [green (12%), light purple (25%), purple line (16%), purple (47%)], leaf: angle [erect (5%), semi erect (9%), horizontal (38%), droopy (47%)], culm: attitude [erect (1%), semi erect (10%), open (28%), spreading (61%)],internodes: colour [green (9%), light gold (12%), purple line (56%), purple (23%)], flag leaf: attitude of blade [erect (4%), semi erect (2%), horizontal (20%), deflexed (75%)] were detected with highest variation in the *O. rufipogon* accessions. For rest of the characters, two and/or three alternative forms were detected in both *O. nivara* and *O. rufipogon*. The neighbour-joining dendrogram constructed based on pair-wise genetic distance calculated from these phenotypic descriptors grouped the genotypes into several small groups. In the two distinct clusters some overlapping between *O. rufipogon* and *O. nivara* were observed due to the presence of some similar morphological descriptors between the two groups. However, majority of *O. rufipogon* accessions were clustered in one group, other than the *O. nivara* groups. *O. rufipogon* accessions were observed to have distinctiveness for morphological characters but *O. nivara* accessions shared the morphological characters with some of the *O. rufipogon* accessions.

### Agronomic traits

**Figure 2:**
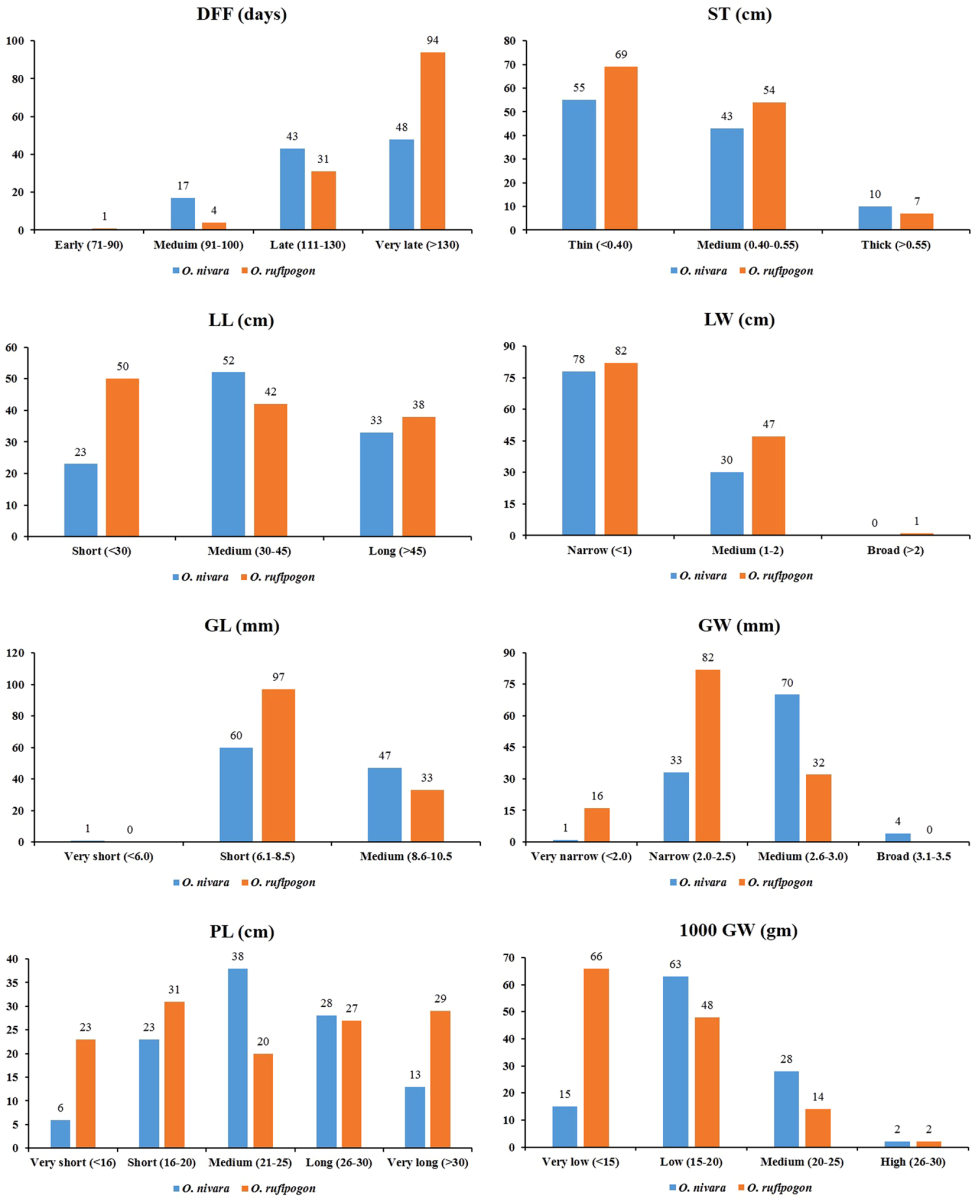
Comparison of agro-morphological traits between and/or within *O. rufipogon* and *O. nivara* accessions.

High level of variation was observed in *O. rufipogon* and *O. nivara* accessions for different agronomic traits. The duration for 50% flowering ranged from 101–159 days among *O. rufipogon* accessions, while it ranged from 101–136 days in *O. nivara* (Supplementary Table S2 and Fig. 2). *O. rufipogon* accessions were medium slender to long slender for their grain characteristics (grain length: 7.08–9.26; grain breadth: 1.76–2.94), but some short bold and short medium grain types (grain length: 4.27–9.46; grain breadth: 1.99–3.25) were detected in the *O. nivara* accessions. Some of the accessions of *O. rufipogon* were observed to be having broader leaf as compared to *O. nivara*. However, no significant variation for leaf length, ligule length, culm diameter, 100 grain weight and panicle length was detected between *O. rufipogon* and *O. nivara* groups. Thin or medium thick stem types were detected in most of the *O. rufipogon* and *O. nivara* accessions. Further, only one *O. rufipogon* accession was identified to have broad leaf (>2 cm). Highest variation was detected for panicle length within *O. rufipogon* and *O. nivara* collections.

### Molecular characterization

#### SSR marker diversity

The surveyed 36 SSR markers revealed the availability of a high level of genetic diversity in *O. rufipogon* and *O. nivara* collections. The markers amplified a total of 144 alleles with an average of 4 per locus (Table 1). RM8020 amplified a highest of 7 alleles, followed by 6 alleles with RM495, RM3866, RM16649, RM336 and RM72. The loci, RM6378, RM422, RM413 and RM3472 could amplify 5 alleles each, while RM423, RM5780, RM3392, RM163, RM528, RM510, RM253, RM547, RM215, RM447, RM8207, RM206, RM5918 and RM2529 showed 4 alleles in the entire set of wild accessions. One locus i.e. RM261 could detect only one allele which differentiated the accessions for its presence or absence. The number of effective alleles for the tested loci ranged from 1.000 to 4.331 for the markers RM261 and RM8020, respectively which was directly related to number of alleles amplified by the markers. Effective allele is the number of alleles amplified with equal frequencies in the entire population. PIC value of a marker determines the effectiveness of diagnostic power for that particular marker in the given set of genotype. In our study, the PIC value for the 36 SSRs varied from 0.428 (RM261) to 0.982 (RM16649). Of the 36 loci, 17 could detect a high PIC value of more than 0.900 and the average PIC value calculated with all the markers used in this study was 0.852. The markers could clearly reveal that expected homozygocity (Ho) of any given loci is inversely proportional to its heterozygocity/genetic diversity (He). RM8020 showed a highest genetic diversity (He) of 0.769 followed by RM16649 (He = 0.746). Similarly, RM244 showed lowest genetic diversity (He = 0.080). However, RM261 distinguishing the accessions with only one allele, could not generate any genetic diversity for the tested set of accessions. The average genetic diversity value detected with the markers was 0.566 and 7 of the loci were detected with a genetic diversity value of more than 0.700. Similar trend was detected for Shannon’s genetic diversity index (I) for all the 36 markers.

**Table 1:** Genetic diversity parameters calculated on SSR data.

Locus	Na	Ne	PIC	Ho	He	I
RM495	6	3.208	0.902	0.31	0.688	1.329
RM10864	3	2.259	0.797	0.441	0.557	0.941
RM3642	3	2.382	0.886	0.419	0.58	0.978
RM6378	5	3.526	0.936	0.282	0.716	1.391
RM423	4	3.193	0.869	0.312	0.687	1.264
RM5780	4	3.055	0.966	0.326	0.673	1.221
RM422	5	2.921	0.863	0.341	0.658	1.17
RM81B	3	1.955	0.783	0.511	0.489	0.835
RM3392	4	2.559	0.881	0.39	0.609	1.021
RM3866	6	2.975	0.915	0.335	0.664	1.227
RM261	1	1	0.428	1	0	0
RM16649	6	3.941	0.982	0.251	0.746	1.525
RM480	3	2.328	0.928	0.428	0.57	0.92
RM413	5	2.524	0.974	0.395	0.604	1.093
RM163	4	3.036	0.903	0.328	0.671	1.219
RM528	4	2.365	0.956	0.422	0.577	0.997
RM510	4	2.091	0.666	0.477	0.522	0.797
RM204	3	1.803	0.927	0.553	0.446	0.767
RM336	6	3.535	0.929	0.281	0.717	1.426
RM253	4	2.421	0.892	0.412	0.587	1.017
RM3404	3	1.712	0.814	0.583	0.416	0.634
RM8020	7	4.331	0.975	0.229	0.769	1.61
RM72	6	3.924	0.958	0.253	0.745	1.492
RM547	4	2.323	0.936	0.429	0.57	1.077
RM215	4	2.133	0.776	0.468	0.531	0.927
RM205	3	2.739	0.802	0.364	0.635	1.046
RM447	4	2.272	0.873	0.438	0.56	1.063
RM244	2	1.086	0.581	0.92	0.08	0.173
RM8207	4	2.19	0.673	0.456	0.543	0.948
RM590	2	1.343	0.532	0.744	0.255	0.423
RM206	4	3.794	0.927	0.262	0.736	1.358
RM287	3	2.869	0.853	0.347	0.652	1.074
RM5918	4	1.767	0.87	0.565	0.434	0.845
RM3472	5	3.73	0.917	0.267	0.732	1.406
RM463	2	1.98	0.871	0.504	0.495	0.688
RM2529	4	1.859	0.943	0.536	0.462	0.821
Mean	4	2.587	0.852	0.433	0.566	1.02
St. Dev	1.331	0.819	0.129	0.172	0.171	0.352

Na: number of alleles; Ne: number of effective alleles; PIC: polymorphism information content; Ho: expected homozygosity; He: expected heterozygosity/Nei’s genetic diversity; I: Shannon’s information index.

The molecular markers were categorized based on their repeat motif. Out of 36 total loci, 17 were with di-nucleotide repeat motif, 13 having tri-nucleotide and 2 having tetra-nucleotide. Further, 4 loci had multi-nucleotide repeat motif. The average number of alleles was highest (4.750) for markers having multi-nucleotide repeat motif followed by tri-nucleotide (4.077), di-nucleotide (3.882) and tetra-nucleotide (3.000) (Table 2). However, the average PIC value was highest with tetra-nucleotide repeat motif and was lowest in case of tri-nucleotide repeat motif. But, the genetic diversity value (He) was 0.586 (highest) for di-nucleotide repeat motif followed by 0.566 (multi-nucleotide repeat motif), 0.544 (tri-nucleotide repeat motif) and 0.536 (lowest) for tetra nucleotide repeat motif.

**Table 2:** Genetic diversity parameters of SSRs based on their repeat motif.

Repeat Motif	Na	Ne	PIC	Ho	He	I
Di-nucleotide	3.882	2.535	0.868	0.413	0.586	1.012
Tri-nucleotide	4.077	2.562	0.821	0.454	0.544	1.027
Tetra-nucloetide	3	2.173	0.914	0.463	0.536	0.843
Multi-Nucleotide	4.75	3.094	0.854	0.433	0.566	1.123

Na: number of alleles; Ne: number of effective alleles; PIC: polymorphism information content; Ho: expected homozygosity; He: expected heterozygosity/Nei’s genetic diversity; I: Shannon’s information index.

#### Rare and unique alleles

The alleles which were amplified in less than 5% of the entire population for any given loci were considered as rare alleles^31^. A total of 30 rare alleles were amplified in 19 out of 36 tested loci for all the accessions. The loci RM16649 could amplify a highest of 4 (150 bp in 9 accessions, 160 bp in 8 accessions, 180 bp in 11 accessions and 270 bp in 6 accessions) rare alleles (Supplementary Table S3). Another marker, RM2529 also amplified 4 rare alleles i.e. 150 bp (8 accessions), 180 bp (5 accessions), 200 bp (5 accessions) and 210 bp (2 accessions). Majority of the rare alleles were observed in *O. rufipogon* accessions (cumulative n = 116) than in *O. nivara* accessions (cumulative n = 71). Of these, some of the accessions were detected with more than one rare allele for more than one loci. For instance, AC100141 (*O. rufipogon*) showed two rare alleles of 120 bp for RM336 and 200 bp for RM8020.One *O. nivara* accessions i.e. AC100467 amplified highest number of rare alleles at 4 loci (RM423, RM5780, RM480 and RM253). In case of *O. rufipogon* population, rare alleles were detected in the accessions like AC100474, AC100323, AC100438, AC100439, AC100346 and AC100430 for a highest of 3 loci in different combinations. Further, some of the *O. rufipogon* and *O. nivara* accessions shared similar rare alleles. For example, *O. rufipogon* accessions like AC100279 and AC100323 shared the rare allele of 170 bp of RM495 with the *O. nivara* accessions like AC100450, 100448, AC100477, AC100471, AC100468, AC100297 and AC100338. Similar types of allele sharing between *O. rufipogon* and *O. nivara* were detected for RM16649 (160 bp and 180 bp), RM510 (130 bp), RM336 (120 bp) and RM72 (200 bp).

Unique allele addresses the potentiality of any given loci to detect an accessions with any distinct amplicon that is different from the entire population. In our study, a total of 5 unique alleles were identified with the tested set of loci. Interestingly, one *O. nivara* accession, AC100313, was detected with the maximum of 3 unique alleles of 250 bp (RM423), 280 bp (RM422) and 500 bp (RM3392) (Supplementary Table S4). Further, another *O. nivara* accession (AC100016) showed uniqueness for RM510 with an amplicon of 400 bp and *O. rufipogon* accession (AC100485) was detected to amplify a unique allele for RM3866 (450 bp).

#### Allele sharing between *O. rufipogon* and *O. nivara* population

Out of total 36 loci tested, 34 could amplify 83 common alleles between *O. rufipogon* and *O. nivara* accessions. The locus, RM72 was detected with a highest of 5 (100 bp, 130 bp, 150 bp, 160 bp, 180 bp and 200 bp) common alleles between both the populations (Supplementary Table S5). Further, 7 of the loci (RM495, RM6378, RM16649, RM336, RM447 and RM206) amplified 4 common alleles each. However, RM528 and RM8020 clearly distinguished *O. rufipogon* accessions from *O. nivara* accessions for their allele size. For example, RM528 amplified 280 bp, 290 bp, 300 bp and 1150 bp allele in *O. rufipogon* but it amplified 240 bp, 250 bp and 260 bp in *O. nivara*. Further, total of 47 unique amplicon were detected in the entire *O. rufipogon* population which were distinct from the 39 unique amplicons of *O. nivara*. Markers like RM10864, RM81B, RM261, RM72, RM447, RM590, RM206 and RM287 could not differentiate *O. rufipogon* from those of *O. nivara* based on their allele size.

#### Genetic variation between *O. rufipogon* and *O. nivara* populations

Separate analysis was carried out to understand the SSR level variation between *O. rufipogon* and *O. nivara* accessions. A total of 256 alleles were detected in *O. rufipogon* collection, of which 127 alleles (average alleles per locus = 3.528) were amplified in accessions collected from Odisha and 129 (average alleles per locus = 3.583) in those collected from West Bengal (Table 3). For *O. rufipogon* accessions, the expected homozygosity (Ho) was marginally higher in the Odisha collection (0.488) than in West Bengal collection (0.479). However, the genetic diversity value was higher in the accessions collected from West Bengal (0.516) as compared to Odisha (0.507). A polymorphism percentage (%P) of 97.220 was observed both in case of Odisha and West Bengal accessions.

**Table 3:** Genetic variation between *O. rufipogon* and *O. nivara* populations.

	Diversity parametes	N	Na	Na*	Ne	I	Ho	He	Nm	%P
*O. rufipogon*	Odisha	60	127	3.528	2.324	0.898	0.488	0.507	0.064	97.220
	West Bengal	68	129	3.583	2.367	0.930	0.479	0.516	0.073	97.220
	Mean	64	128	3.556	2.345	0.914	0.484	0.512	0.068	97.220
*O. nivara*	Odisha	44	114	3.167	2.144	0.821	0.524	0.470	0.049	91.670
	West Bengal	64	127	3.528	2.273	0.884	0.498	0.497	0.055	94.440
	Mean	54	121	3.347	2.208	0.853	0.511	0.484	0.052	93.055

N: number of accessions:;Na: total number of alleles; Na*: average number of alleles; Ne: number of effective alleles; I: Shannon’s information index; Ho: expected homozygosity; He: expected heterozygosity/Nei’s genetic diversity; Nm: gene flow; %P: polymorphism percentage.

A similar trend of variation was observed for *O. nivara* accessions collected from Odisha and West Bengal. A total of 114 alleles and 127 were amplified in the *O. nivara* accessions collected from Odisha (average of 3.167 alleles per locus) and West Bengal (average of 3.568), respectively. In the *O. nivara* accessions of Odisha the expected homozygosity (Ho) was higher (0.524) than of West Bengal (0.498). In contrast, the genetic diversity (He) present in the West Bengal accessions was higher (0.497) than the accessions collected from Odisha (0.470). Similarly, the West Bengal collection showed higher (94.440) polymorphism percentage (%P) as compared to the counterpart i.e. Odisha (91.670).

Cumulative analysis, combining the accessions of both the states, revealed that *O. rufipogon* accessions were more diverse (He = 0.512) than the *O. nivara* accessions (He = 0.484). Similar trend was observed for number of effective alleles (*O. rufipogon* = 2.345, *O. nivara* = 2.208), Shannon’s diversity index (*O. rufipogon* = 0.914, *O. nivara* = 0.853), and polymorphism percentage (*O. rufipogon* = 97.220%, *O. nivara* = 93.055%). The gene flow (Nm) was also higher in *O. rufipogon* (0.068) as compared to *O. nivara* (0.052).

Further, the analysis of molecular variance (AMOVA) was done to reveal the total existing variation within and between populations (both species and geographical region wise). When we grouped the total *O. rufipogon* population and total *O. nivaraO. nivara* population separately, regardless their collection states, 21% of the total variation was present among population while 79% was within population (Table 4). Secondly, in both the cases for *O. rufipogon* and *O. nivara* collected from two different states, the AMOVA could identify only 2% of variation among population and 98% within population.

**Table 4:** Analysis of molecular variance of wild rice accessions – species wise and geographical area wise.

AMOVA based on species			MS	EV	%
Source	df	SS			
Among Populations	1	656.085	656.085	2.737	21%
Within Populations	474	4853.848	10.24	10.24	79%
Total	475	5509.933	—	12.977	100%
AMOVA of rufipogon based on geographical area					
Among Populations	1	33.58	33.58	0.184	2%
Within Populations	254	2584.283	10.174	10.174	98%
Total	255	2617.863	—	10.358	100%
AMOVA of nivara based on geographical area					
Among Populations	1	34.737	34.737	0.236	2%
Within Populations	214	2156.592	10.078	10.078	98%
Total	215	2191.329	—	10.314	100%

df: degree of freedom; SS: Sum of squares; MS: Mean squares; EV: Estimated variance (p < 0.001); %: percentage of variation.

#### Genetic diversity, relationship and population structure

Pair-wise genetic distance is the measure to identify the underlying genetic relationship among the genotypes. The pair-wise genetic distance calculated for all the accessions with 36 SSR markers ranged from 0.148 [between AC100424 and AC100425 (both *O. nivara* accessions collected from 24 Parganas of West Bengal)] to 2.551 [between AC100030 (*O. rufipogon* from Kalahandi of Odisha) and AC100313 (*O. nivara* from Midnapore of West Bengal)] with an average of 0.852.

**Figure 3:**
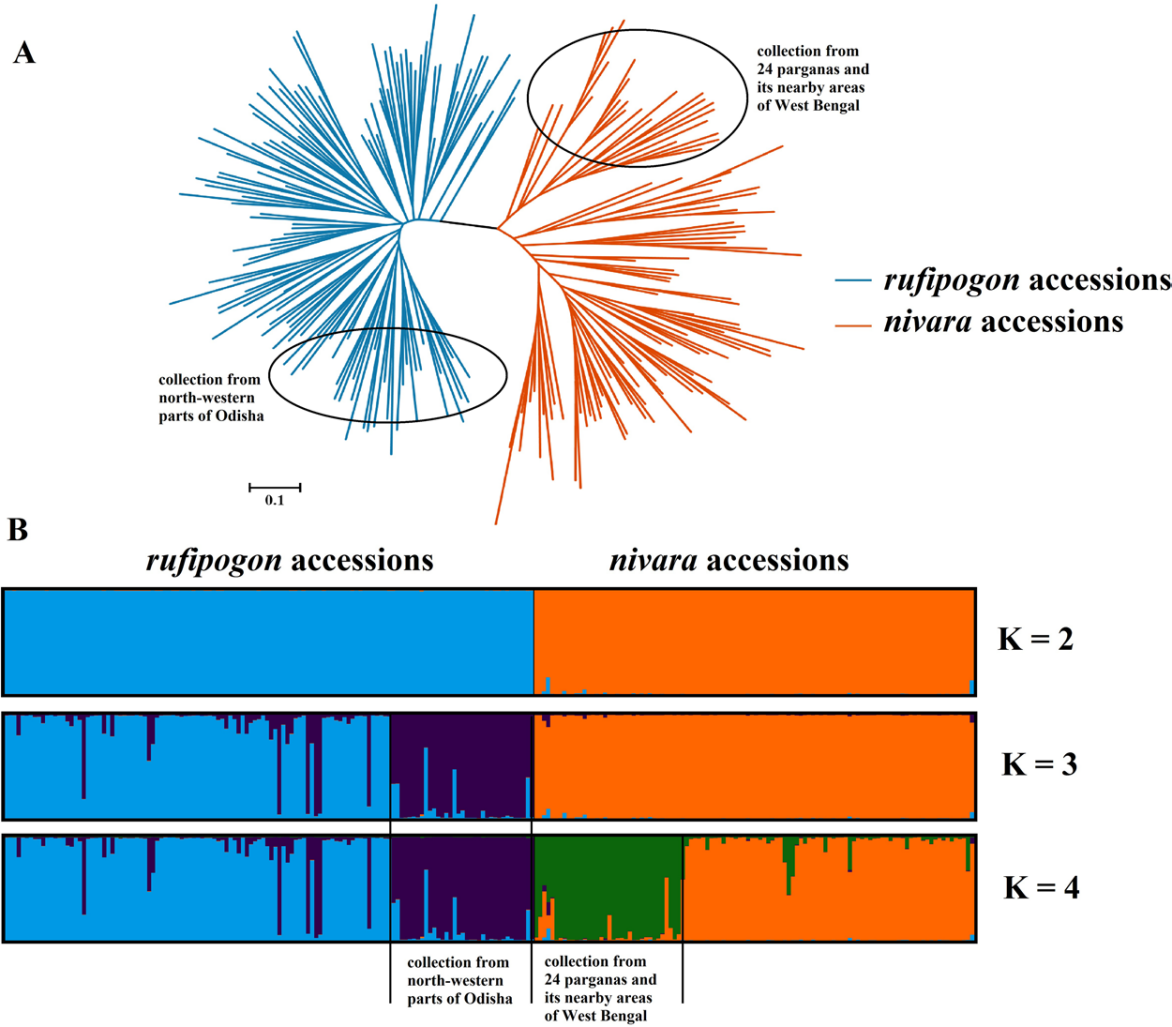
Clustering, speciation differentiation between *O. rufipogon* and *O. nivara* based on neighbor-joining tree and population structure analysis of the wild rice accessions with hyper variable SSR markers.

The neighbour-joining dendrogram based on pairwise genetic distance measure between any two pair was constructed to understand the genetic relationship among the accessions at molecular level. The dendrogram could clearly separate *O. rufipogon* and *O. nivara* accessions in to two distinct clusters. Further, the *O. rufipogon* accessions were observed to be closely related to each other with 4 major sub-clusters and some minor sub-clusters. But, the *O. nivara* accessions were distantly associated generating several sub-cluster (Fig. 3A). The *O. rufipogon* accessions were grouped together having less distance from each other as compared to the *O. nivara* accessions. Two distinct sub-clusters, both in *O. rufipogon* and *O. nivara* clusters were detected.

The population structure analysis revealed highest value of 959.28 at K = 2, suggesting that the entire collection could be divided into two distinct sub-populations i.e. SP1 and SP2 (Fig. 3B). SP1 contained all the *O. rufipogon* accessions with a membership percentage of 54.6, while all the *O. nivara* accessions were in SP2 with a membership percentage of 45.4 (Table 5). The average genetic distance and *F*_ST_ of the *O. rufipogon* accessions of SP1 were 0.500 and 0.324, respectively. Similarly, the average genetic distance of SP2 (*O. nivara* sub-population) was 0.520 with a *F*_ST_ value of 0.264. The distinctiveness of *O. rufipogon* and *O. nivara* in separate sub-population was similar to the observations in neighbour-joining tree.

**Table 5:** Population statistics of detected sub-population clusters.

At different ‘K’	Sub-population	Membership (%)	FST	Average distances
K = 2	SP 1	54.6	0.324	0.5
	SP 2	45.4	0.264	0.52
K = 3	SP 1	37.8	0.348	0.498
	SP 1-1	16.8	0.223	0.489
	SP 2	45.4	0.179	0.513
K = 4	SP 1	37.8	0.225	0.512
	SP 1-1	16.8	0.268	0.486
	SP 2	30.7	0.325	0.473
	SP 2-2	14.7	0.364	0.456

SP: Sub population.

Moreover, at K of 3 the *O. rufipogon* sub-population was further divided into two separate groups i.e. sub-population 1–1 and sub-population 1–2 with a membership percentage of 37.8 and 16.8, respectively. Further increase of K to 4 divided the *O. nivara* accessions (SP2) to sub-population 2–1 (membership percentage of 30.7) and sub-population 2–2 (membership percentage of 14.7). This observations were in line with the neighbour-joining tree where the separated group in population structure analysis could clearly be identified.

#### Genetic relationship of rice accessions based on their geographic districts of collection

The genetic diversity measures were calculated based on the accessions collected from a particular district. The district from which < 5 accessions were available were not included in the analysis. The average number of allele for any district ranged from 3.917 (Bardhhaman) to 1.729 (Mayurbhanj) (Supplementary Table S6). Similarly, the number of effective allele ranged from 2.697 for Bardhhaman to 0.028 for Mayurbhanj. The locally common allele for a particular district with a frequency of >25% was highest (0.389) for Bankura district, whereas it was lowest (0.028) for Mayurbhanj. However, the accessions collected from Bardhaman district showed highest diversity of 0.569 followed by Midnapore (0.548) and Mayurbhanj district was lowest counterpart with a diversity value of 0.349 for its wild accessions. The analysis of molecular variance revealed that there existed 9% of the total variation among the districts and rest 91% were within them (Supplementary Table S7).

**Figure 4:**
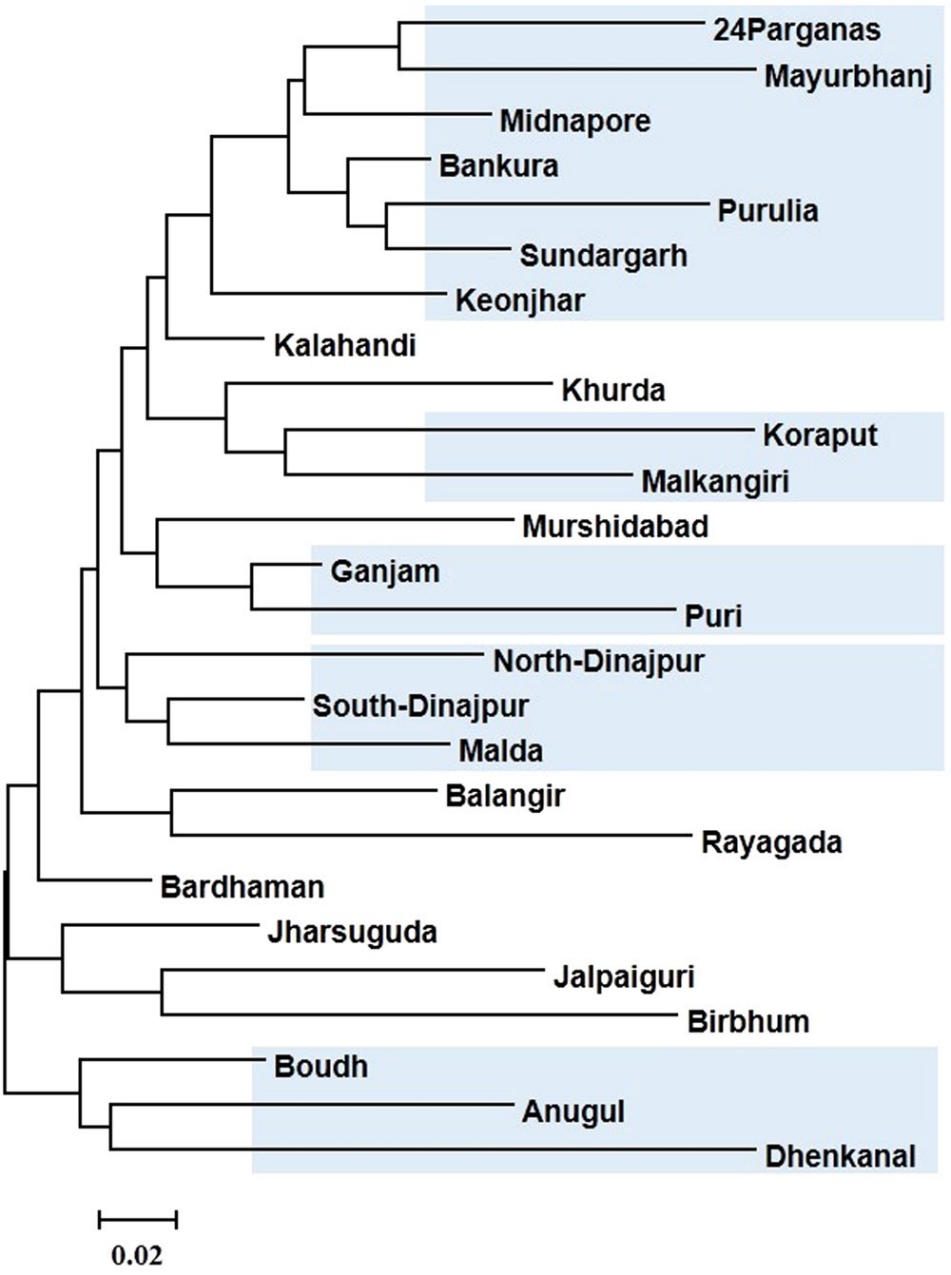
Neighbour-joining dendrogram showing relationship and differences of wild rice accessions based on different districts of collection.

The entire collection of wild accessions i.e. *O. rufipogon* and *O. nivara* were grouped based on their districts of collection from both the states to understand the genetic relationship among different districts. The neighbour-joining dendrogram constructed based on pair-wise genetic distance between districts grouped the 26 districts into two major clusters. The districts like Boudh, Anugul and Dhenkanal present in the middle part of Odisha were separated from rest of the districts and were grouped in a different cluster (Fig. 4). Critical observations showed that most of districts which share the adjacent geographical areaswere grouped together. For example, close relationship was observed between Malda, South-Dinajpur and North-Dinajpur of West Bengal, Puri and Ganjam of Odisha, Koraput and Malkangiri of Odisha. Further, the districts like Sundargarh, Keonjhar and Mayurbhanj of Odisha were closely associated with their nearby West Bengal districts like 24Parganas, Midnapore and Bankura were grouped together.

## Discussion

The aim of the present study was to harness the potential of morphological and molecular variation available within and between two wild rice populations i.e. *O. rufipogon* and *O. nivara* collected from West Bengal and Odisha, two major rice growing states of eastern India. In these states, in general, the farmers are marginal having small land holdings and employ traditional practices. Rice cultivation in these areas is quite challenging as irrigated area is less and proportion of rainfed rice is high. Under this scenario, biotic and abiotic stresses are of common occurrence as most of the area is classified as uplands or low lands. However, the potential to enhance productivity and production in eastern India is high as a number of wild rice accessions are still available in these states and thorough characterization of them could lead to identification of suitable material for rice improvement. Since both *O. rufipogon* and *O. nivara* are closely related and also sharea close relationship with the Asian cultivated rice (*O.sativa)*, the characterization of the diversity in them could lead to understand their dynamic relationship.

At morphological level, as expected, a high level of variation was detected in both *O. rufipogon* and *O. nivara* accessions. Awn, a specificfeature of wild forms, wasdetected in all the accessions. Interestingly, high variation (4 alternative forms for each trait) was detected for traits like basal leaf sheath colour, leaf angle, culm attitude, internode colour and flag leaf attitude in both wild forms but the frequencies varied between the species. For example, in *O. nivara*, the green basal leaf sheath was predominant (52%), while majority (47%) of the *O. rufipogon* accessions were having purple basal leaf sheath. The dendrogram constructed based on the morphological traits clearly demarcates *O. rufipogon* and *O. nivara* into two distinct classes.

The wide variation observed in *O. rufipogon* and *O. nivara* for different agronomic traits provide us signs related to their ecological adaptation. While majority of the *O. rufipogon* and *O. nivara* accessions are of late duration, the medium duration accessions are of interest as they can lead us to understand the mechanisms involved in tolerance to drought and photo period sensitivity and the underlying genes involved. Earlier studies reported, fine type of grain in *O. rufipogon* and bold type in *O. nivara*^32^. In the accessions studied, variation for grain type was observed in both wild forms with medium slender and long slender grains observed in *O. rufipogon* while majority of the accessions of *O. nivara* are either short bold or short medium type. All the accessions under study are with weak culm and one of the major drawbacks in wild rices is culm thickness and for this reason, wild rices are of spreading type.

Marker technologies can dissect variation efficiently at molecular level and the 36 highly variable SSR markers employed in the study could amplify a total of 144 alleles, a value much higher than the number of alleles detected in wild rice population of Uttar Pradesh and Bihar of India^33^ where a total of 106 alleles were detected. Further, Singh and his colleagues^24^ could detect an average of 2.4 alleles per locus with 25 SSR markers in wild rice collection of Indo-Gangetic Plains of India whereas, we could detect an average of 4 alleles per locus. Instead of using random SSRs, targeting the hyper variable regions of rice chromosome to exploit their genetic diversity would have an edge over the random SSRs. Except one locus i.e. RM261, high allelic variationwas seen with 35 markers used and from the results it can be suggested that the present set of markers used in this study can serve as a reference set for genetic diversity studies in rice. This observation was further justified as these markers showed high resolving power with PIC values ranging from 0.428 for RM261 to 0.982 for RM16649 and 17 out of 36 loci had a PIC value of more than 0.900. The average PIC value in our study (0.852) was higher than the value reported earlier in wild rice of Indo-Gangetic plains of India (0.790)^24^, wild rice collection of UP and Bihar of India (0.5247)^32^ and Indian rice mini-core collection (0.671)^34^.

As expected, the diversity detected in *O. rufipogon* and *O. nivara* accessions of eastern India (average He = 0.577) was higher than the diversity reported in Indian rice hybrids (He = 0.432)^35^, Thai rice lines (He = 0.436)^36^ and rice germplasm of DPRKorea (He = 0.340)^37^. Further, this set of wild accessions were more diverse than the wild rice collections of eastern Indo-Gangetic plains^24^, while it was quite comparable with the wild rice collection from UP and Bihar of India^33^. As the Sundarbans delta of West Bengal and western part of Odisha are considered as two major biodiversity hot spotsfor different flora and fauna, the wild rice accessions of these regions and nearby areas are expected to display higher amount of genetic diversity^38,39^. Further, predominance of tribal communities who still prefer traditional land races and traditional farming practices in these two states (Odisha and West Bengal) as compared to other states could be a reason for low level of genetic erosion in these areas.

Interestingly, more than 20% of the total amplified alleles were identified as rare alleles. This observation confirms the earlier reports that wild rices could be preferred over cultivated varieties/landraces for mining valuable alleles for improvement of rice. The rare alleles may be associated with special traits which could be highly valuable in breeding programmes after their validation. In our study, of the five unique alleles detected, three were detected in a single *O. nivara* accession i.e. AC100313. Since *O. nivara* it the closer to *O.sativa*, genome sequencing of this particular *O. nivara* accession could unravel its genetic constitution. Different QTLs associated with drought tolerance were mapped on rice chromosome 2 and 3 and since *O. nivara* is adapted to upland conditions, the unique alleles identified on these chromosomes are of great interest.

Of the thirty six locitested, two loci could differentiate *O. rufipogon* from *O. nivara* effectively with distinct allelic variation between the two populations. The allele sharing between *O. rufipogon* and *O. nivara* was detected to be high (57.64%), revealing close genetic association between them. Close relationship between these wild forms of eastern India might be due to a common ancestry and/or regular gene flow between them. This observation is similar to the reports of Banaticla-Hilarioand colleagues^40^ who also reported more than 50% of allele sharing between *O. rufipogon* and *O. nivara*. As expected, *O. rufipogon* accessions were more diverse (He = 0.512) as compared to *O. nivara* (He = 0.484)^40^. The higher level of gene flow (Nm = 0.068) detected in *O. rufipogon* population can be attributed to its higher out pollination rates.

Though there was extensive allele sharing, species differentiation (21% variation between populations of *O. rufipogon* and *O. nivara*) detected in the study is similar to the results on the collections from Indo-Gangetic plains of India^24^ and Vientiane region of Laos^23^. However, we could not detect any regional differences between the collections of West Bengal and Odisha which could be due to their close geographical location or due to diversification at a place and subsequent spread to the other region. The Bayesian model based clustering showed clear molecular divergence of *O. rufipogon* and *O. nivara* into two separate and distinct populations at K = 2^23,40^. Similar to nuclear genome variation^25,41^, organelle level variation i.e. chloroplast, mitochondria, between *O. rufipogon* and *O. nivara* was reported earlier^42,43^. Another interesting observation in our study was the absence of admixtures in the entire population which is in contrast to the report of Vaughan and his group^44^ who reported abundant presence of introgressed/intermediate forms in the Asian gene pool. The availability of true to type *O. rufipogon* and *O. nivara* accessions will be of immense value to the future rice improvement programs. However, further studies are needed to ascertain the allelic similarity/diversity between the wild and cultivated forms.

One of the basic questions is how to treat *O. rufipogon* and *O. nivara* as separate groups on the basis of variation within and between the populations. Rather than analyzing the sub-populations (already identified at K = 2) separately, manually increasing the K value of the previous analysis was preferred^45^. Interestingly, at K = 3, the *O. rufipogon* accessions collected mostly from North-western regions of Odisha, formed an independent group and at K = 4, the *O. nivara* accessions from 24 Parganas and its adjacent districts of West Bengal formed a separate group. From the neighbour-joining dendrogram of different districts, it can be observed that the collections from adjoining districts that a share a common boundary, were grouped together. Moreover, a close association was observed between a group of districts of West Bengal comprising 24 Parganas and its nearby districts and the North-western districts of Odisha suggesting similarity over a broad geographical area. In Odisha and West Bengal, rice is generally grown under rainfed conditions and the region has both uplands and low lands, the habitats suitable for the growth of *O. nivara* and *O. rufipogon* respectively. Zheng and Ge^21^ suggested that domestication, natural selection and gene flow events must have led to the fixation of different genetic constitutions of *O. rufipogon* and *O. nivara* accessions.

Banaticla-Hilarioand colleagues^40^ studied the differentiation of *O. rufipogon* and *O. nivara* and compared the sympatric and non-sympatric populations. In their analysis, when *O. meridionalis* was added to the other two groups (*O. rufipogon* and *O. nivara*) of Southeast Asia, only 10% variation was detected among the sympatric populations. But, in our case, where the populationsare non-sympatric, we could detect 21% variation among species, thus clearly demonstrating the level of differentiation was non-sympatric populations than in their sympatric counterparts. This result provides some substance to the first domestication events and suggest thepossibility of a common ancestor for these two wild ancestors of rice.

Origin of *O. nivara* has always been a debatable issue since 1960s and it was considered as a distinct species^7,10^ while it was treated as a habitat shift related speciation from *O. rufipogon*^4^. The observation of highly diverse accessions in both *O. nivara* and *O. rufipogon*, with little sharing of common alleles among them indicates that these two forms can be treated as diverse forms. Normally, description and differentiation between *O. rufipogon* and *O. nivara* has been focused with sympatric population of different locations in earlier studies. However, population genetic structure of these two wild relatives in a non-sympatric population has not been exploited much. From the results obtained in the present study where genetic variation between *O. rufipogon* and *O. nivara*, a non-sympatric population of eastern India was focused, it is interesting to differentiate the two groups in separate cluster at minimal model based analysis. Though there is evidence for conservation of some of the morphological traits and sharing of allelic uniqueness between the two groups, existence of them in a non-sympatric manner and their clear differentiation in alternative groups provides insights to the idea that *O. rufipogon* and *O. nivara* could be treated as different species. This is possible either through (i) accessions of both could have been evolved separately or (ii) one could have evolved from the other and fixation of genetic constituent thereafter according to agro-ecological condition leading to some of the intermediate and alternative forms due to the ecological shift to form an annual that lack in photosensitivity trait and with a curtailed duration.

## Methods

### Germplasm collection and agro-morphological characterization

**Figure 5:**
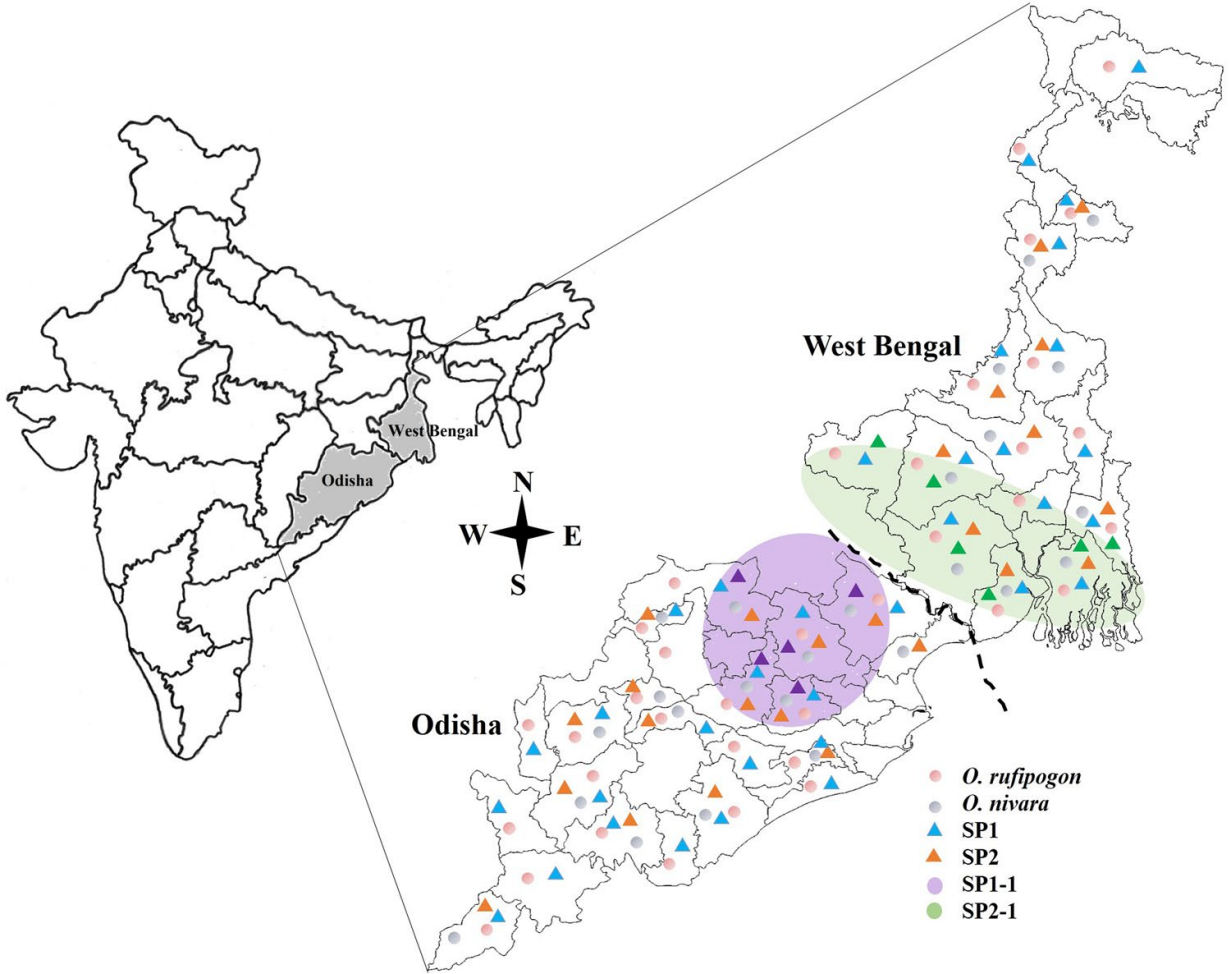
Sampling sites of *O. rufipogon* and *O. nivara* from different areas of Odisha and West Bengal and information on population structure. The Map was hand drawn, scanned and edited with Photoshop image editor 7.0.1.

A total of 130 *O. rufipogon* and 108 *O. nivara* wild rice accessions were collected from various areas of Odisha and West Bengal (Supplementary Table S8 and Fig. 5). Further, these collected wild rice accessions were categorized according to their districts of collection of both the states. All the accessions were grown in individual pots in net-houses of National Rice Research Institute, Cuttack, Odisha during 2014–16 under controlled environmental conditions.

Twenty-two phenotypic characters^46^ (Supplementary Table S1) were measured at different growth stages following the guidelines of International Rice Research Institute for two consecutive years i.e. 2015–2016. Further, data was recorded on agronomic traits like heading duration, culm length, culm diameter, culm number, leaf length, leaf width, ligule length, panicle length, grain length, grain breadth, L/B ratio and 100 grain weight were recorded for each accession^47^.

### Isolation of genomic DNA, PCR assay and genotyping

Fresh leaf samples were harvested from one-month young seedlings and genomic DNA was extracted from each wild rice accessions (both *O. rufipogon* and *O. nivara*) following a modified cetyltrimethylammonium bromide extraction protocol^48^. Thirty-six SSR loci amplifying the hyper variable regions^49^, three from each of 12 chromosomes of rice were selected for the genetic diversity analyses. Details of the SSR loci used in the present study are given in Supplementary Table S9. The PCR amplifications were performed in a total reaction volume of 10 μl containing 20 ng genomic DNA, 2.0 picomoles of each forward and reverse primer, 1 μl of 10X buffer (0.1 M Tris pH 9, 0.5 M KCl, 15 mM MgCl2, 0.1% gelatine), 200 μM each of dNTPs and 0.3 U of *Taq* DNA polymerase. The PCR condition was an initial denaturation at 94 °C for 5 min followed by 35 cycles of denaturation at 94 °C for 30 sec, annealing (depending on TM value of primer) at 50–60 °C for 45 sec, extension at 72 °C for 1 min and a final extension of 7 min at 72 °C. The final PCR products were resolved in 2.5% ethidium bromide stained (1 µg/ml) agarose gel. The separated PCR products were visualized under UV light and photographed using Typhoon FLA 7000 fluorescent image analyzer (GE Healthcare Bio- Sciences AB, Uppasala, Sweden). The size of each intense amplified fragment for all SSR loci was determined by comparison with the size standard (100 bp DNA ladder) and scored by incremental numbering from the lowest molecular weight band to the higher molecular weight bands and the genotype matrix was prepared.

### Data analysis

The amplified bands/alleles were scored as present (1) or absent (0) for each genotype and primer combination. The data were entered into a binary matrix and subsequently analyzed using different computer software packages. An allele that was observed in <5% was considered to be rare allele.The polymorphism information content (PIC) for each SSR marker locus was calculated using the formula: ${\rm{PICi}}=1-{\sum }_{j=1}^{n}{(Pij)}^{2}$, where n is the number of marker alleles for marker i and P_ij_ is the frequency of the j^th^ allele of marker I^50^. Genetic diversity parameters viz., number of alleles (Na), effective number of alleles (Ne), expected homozygosity (Ho), Nei’s genetic diversity index/expected heterozygosity (He)^51^ and Shannon Index (I) were evaluated using POPGENE v 1.32 (http://www.ualberta.ca/fyeh) with 1,000 permutations. The dendrogram based on unbiased genetic distances among genotypes was constructed by un-weighted neighbour joining tree employing MEGA 6^52^. Principal coordinate analyses (PCoA) of the accessions with 36 SSR loci were performed based on the simple matching coefficient using the dcenter and eigenvector matrices in the software GeneALEx6^53^ with 1,000 random permutations. The Bayesian model-based clustering analysis of the genotypes was used for determining the optimal number of genetic clusters found among rice varieties using the STRUCTURE software^54^ with 1,00,000 burn-in (iteration) periods and 1,00,000 Markov Chain Monte Carlo (MCMC) replicates with ten independent runs (K) ranging from 1 to 10. The ΔK based on the change in the log probability of the data between successive K values^55^ was estimated using the software program Structure Harvester v6.0^56^ and population clusters were produced by the software Structure Plot^57^ (http://btismysore.in/strplot). Moreover, genotypes were further grouped based on their collection by geographical location (districts) and the genetic diversity parameters of the genotypes within each district were determined using POPGENE v 1.32. The genetic variation within and among the populations was calculated by the procedure of AMOVA (Analysis of Molecular Variance) using the software GeneALEx6^53^.

## Electronic supplementary material

Supplementary Table S1-S9
