# Supplementary material for: Morphological and molecular dissection of wild rices from eastern India suggests distinct speciation between O. rufipogon and O. nivara populations
Source: Sci Rep. 2018 Feb 9;8:2773. doi: 10.1038/s41598-018-20693-7 (PMC5807453; doi:10.1038/s41598-018-20693-7)
Supplement: Supplementary file 1 — Supplementary Table S1-S9 [file 41598_2018_20693_MOESM1_ESM.doc]

- Title

*Morphological and molecular dissection of wild rices from eastern India suggests distinct speciation between O. rufipogon and O. nivara populations*

• Author(s)

Rashmita Samal†

rashmitasamal@gmail.com

Pritesh Sundar Roy†

priteshroy1@gmail.com

Auromira Sahoo

auromira2010@gmail.com

Meera Kumari kar

meera_kar@hotmail.com

Bhaskar Chandra Marndi

bcmarndicrri@gmail.com

Gundimeda Jwala Narasimha Rao*

gjnrao@gmail.com

• The affiliation(s)

1 Divisionof Crop Improvement, National Research Institute, Cuttack -753006, India

* Corresponding author

†Contributed equally to the work

• Full address for correspondence

Dr. GJN Rao

National Research Institute,

Cuttack -753006, India

E-mail: gjnrao@gmail.com

| **Sl.No.** | **Qualitative Character** | **Abbreviation** | ***O. nivara*** | ***O. rufipogon*** |
| --- | --- | --- | --- | --- |
| 1 | Flag leaf : Attitude of blade | FLA | P | P |
| 2 | Culm: Attitude | CA | P | P |
| 3 | Stem : Thickness | ST | P | P |
| 4 | Panicle :Awns | PA | M | M |
| 5 | Panicle: Colour of awns | PCA | P | P |
| 6 | Panicle: Curavate of main axis | PMA | P | M |
| 7 | Spiklet: Colour of tip of the lemma | SCL | P | P |
| 8 | Panicle: Exertion | PE | P | P |
| 9 | Leaf : Anthocyanine colouration | LA | P | P |
| 10 | Leaf : Intensity of green colour | LI | P | P |
| 11 | Panicle : Secondary branching | PSB | P | M |
| 12 | Leaf: Auricles color | CAUR | P | P |
| 13 | Basal leaf : Sheath colour | BLS | P | P |
| 14 | Leaf: Pubescence of blade surface | LPB | P | P |
| 15 | Leaf: Angle | LAG | P | P |
| 16 | Leaf : Colour of ligule | CL | P | P |
| 17 | Leaf: Shape of ligule | LSL | M | M |
| 18 | Collar : Colour | CC | P | P |
| 19 | Leaf: Auricles | LAR | M | M |
| 20 | Internodes : Colour | INC | P | P |
| 21 | Panicle: Attitude of branches | PAB | P | P |
| 22 | Spikelet : Colour of the stigma | CSTG | P | P |
| **Total number of traits which showed variation** | | | **19** | **17** |

Supplementary Table S1: Morphological traits used in the present study for diversity analysis of wild rice accessions. M: monomorphic; P: polymorphic

| ***O. rufipogon*** | | | | | |
| --- | --- | --- | --- | --- | --- |
| **Variable** | **Max** | **Min** | **Mean** | **Std Dev.** | **Std Err** |
| DFF | 159 | 101 | 140 | 12.90 | 1.13 |
| GL | 9.26 | 7.08 | 8.16 | 0.43 | 0.04 |
| GB | 2.94 | 1.76 | 2.30 | 0.26 | 0.02 |
| LB | 4.68 | 2.70 | 3.58 | 0.42 | 0.04 |
| GW | 2.65 | 0.86 | 1.58 | 0.36 | 0.03 |
| LL | 68.70 | 13.89 | 36.51 | 12.84 | 1.13 |
| LW | 4.07 | 0.57 | 0.95 | 0.36 | 0.03 |
| LGL | 3.96 | 0.66 | 1.93 | 0.79 | 0.07 |
| CL | 176.33 | 45.98 | 108.45 | 33.10 | 2.90 |
| CN | 98 | 7 | 36 | 16.04 | 1.41 |
| CD | 0.56 | 0.20 | 0.39 | 0.08 | 0.01 |
| PL | 38.43 | 12.57 | 24.19 | 7.02 | 0.62 |
| ***O. nivara*** | | | | | |
| DFF | 136.00 | 101.25 | 120 | 7.61 | 0.73 |
| GL | 9.46 | 4.27 | 8.36 | 0.61 | 0.06 |
| GB | 3.25 | 1.99 | 2.57 | 0.24 | 0.02 |
| LB | 4.42 | 2.03 | 3.28 | 0.36 | 0.03 |
| GW | 2.77 | 1.13 | 1.94 | 0.32 | 0.03 |
| LL | 72.04 | 13.37 | 41.36 | 14.39 | 1.38 |
| LW | 1.73 | 0.56 | 0.88 | 0.23 | 0.02 |
| LGL | 3.34 | 0.60 | 1.75 | 0.62 | 0.06 |
| CL | 160.00 | 31.74 | 89.28 | 30.52 | 2.94 |
| CN | 128.60 | 19.00 | 63.50 | 27.92 | 2.69 |
| CD | 0.64 | 0.24 | 0.41 | 0.09 | 0.01 |
| PL | 35.96 | 11.60 | 24.53 | 5.38 | 0.52 |

Supplementary Table S2: Variation for different agronomic traits among the wild rice accessions. DFF: days to 50% flowering; GL: grain length; GB: grain breadth; LB: length/breadth ration; GW: grain weight; LL; leaf length; LW: leaf width; LGL: ligule length; CL: culm length; CN: culm number; CD: culm diameter; PL: panicle length

| **Sl.No** | **Marker** | **Rare allele** | **Accessions** |
| --- | --- | --- | --- |
| 1 | RM495 | 170 bp  200 bp | 100450,100448, 100477, 100471, 100468, 100297, 100338, *100279, 100323*  *100438, 100434, 100480, 100474, 100472, 100409, 100493, 100401, 100038* |
| 2 | RM423 | 280 bp | 100482, 100477, 100475, 100468, 100467, 100463, 100313, 100367 |
| 3 | RM-5780 | 120 bp  200 bp | *100401, 100352, 100279, 100170, 100002, 100317, 100149, 100159, 100310, 100154, 100093*  100486, 100452, 100431, 100423, 100482, 100467, 100463 |
| 4 | RM3866 | 390 bp | *100439, 100473, 100346, 100308, 100323* |
| 5 | RM16649 | 150 bp  160 bp  180 bp  270 bp | *100434, 100048, 100041, 100013, 100433, 100155, 100273, 100324, 100133*  *100435, 100470, 100493, 100491, 100324, 100169,* 100475, 100386  *100456, 100434, 100465, 100030, 100133, 100174, 100159, 100263,* 100431, 100033, 100392  *100284, 100047, 100160, 100314, 100282, 100034* |
| 6 | RM 480 | 280 bp | 100450, 100467 |
| 7 | RM 413 | 150 bp | 100447, 100474 |
| 8 | RM163 | 170 bp | *100430, 100488, 100455, 100449, 100444, 100442, 100461, 100015, 100019, 100457* |
| 9 | RM528 | 260 bp  1150 bp | 100452, 100451, 100468, 100313  *100446, 100438, 100474, 100346, 100001, 100013, 100030, 100156* |
| 10 | RM510 | 130 bp | 100175, 100397, *100430, 100439, 100438, 100483, 100170, 100323, 100036* |
| 11 | RM204 | 150 bp | *100034, 100168, 100457, 100309, 100041, 100174, 100176* |
| 12 | RM336 | 120 bp  200 bp | 100471, 100353, 100008, *100430, 100439, 100436, 100141, 100017, 100093*  100360, 100269, 100304, 100021, 100023,100358, 100367 |
| 13 | RM253 | 290 bp | 100467, 100463, 100450, 100422 |
| 14 | RM3404 | 210 bp | 100011, 100370 |
| 15 | RM8020 | 200 bp  260 bp | *100141, 100343, 100054, 100017, 100005, 100018,*  *100483, 100160* |
| 16 | RM72 | 200 bp | 100453, *100487* |
| 17 | RM8207 | 160 bp | *100449, 100446, 100444, 100343, 100054, 100350, 100093* |
| 18 | RM5918 | 300 bp | 100431, 100313, 100175, 100021 |
| 19 | RM2529 | 150 bp  180 bp  200 bp  210 bp | 100423, 100008, 100120, 100043, 100016, 100416, 100012, 100380  100022, 100007, 100389, 100406, 100185  *100165, 100174, 100143, 100263, 100171*  100332, 100154 |

Supplementary Table S3: List of rare alleles detected by different SSR markers in the wild rice accessions. Accessions in italics are *O.rufipogon*

| **Sl.No** | **Marker** | **Unique allele** | **Accession** |
| --- | --- | --- | --- |
| 1 | RM423 | 250 bp | AC-100313 *(O. nivara)* |
| 2 | RM422 | 280 bp | AC-100313 *(O. nivara)* |
| 3 | RM3392 | 500 bp | AC-100313 *(O. nivara)* |
| 4 | RM3866 | 450 bp | AC-100485 *(O. rufipogon)* |
| 5 | RM510 | 400 bp | AC-100016 *(O. nivara)* |

Supplementary Table S4: Different unique alleles amplified by SSRs in the wild rice collection

| Sl.No | Marker | Common allele (bp) | Uncommon allele (bp) | |
| --- | --- | --- | --- | --- |
|  |  |  | *O. rufipogon* | *O. nivara* |
| 1 | RM 495 | 140, 150, 170, 500 | 200 | 280 |
| 2 | RM 10864 | 200 , 220, 350 | - | - |
| 3 | RM 3642 | 170 , 180 | 200 | 190 |
| 4 | RM 6378 | 120 , 150 , 190, 200 | 180 | - |
| 5 | RM 423 | 290 , 300 | 310 , 550 | 250 , 280 |
| 6 | RM 5780 | 190 | 120, 140, 160 | 150, 180, 200 |
| 7 | RM 422 | 390, 400, 410 | - | 280 |
| 8 | RM 81 B | 120, 130, 150 | - | - |
| 9 | RM 3392 | 180, 190, 200 | - | 500 |
| 10 | RM 3866 | 150, 160, 190 | 300, 390, 450 | - |
| 11 | RM 261 | 130 | - | - |
| 12 | RM 16649 | 160, 180, 200, 220 | 150, 270 | 230, 250 |
| 13 | RM 480 | 200 | 190, 220 | 180 230 |
| 14 | RM 413 | 80 | 90, 100 110, 150 | 50 |
| 15 | RM 163 | 130, 150, 190 | 170 | - |
| 16 | RM 528 | - | 280, 290, 300, 1150 | 240, 250, 260 |
| 17 | RM 510 | 120, 130, 350 | - | 400 |
| 18 | RM 204 | 130 | 100 | 140, 450 |
| 19 | RM 336 | 120, 150, 180, 190 | - | 170, 200 |
| 20 | RM 253 | 120 | 280, 290, 300, 1150 | 240, 250, 260 |
| 21 | RM 3404 | 190, 200 | - | 210 |
| 22 | RM 8020 | - | 150, 200, 210, 260 | 180 |
| 23 | RM 72 | 100, 130, 150, 160, 180, 200 | - | - |
| 24 | RM 547 | 250, 300 | 200, 290 | 230 |
| 25 | RM 215 | 150 | 600 | 160, 170, 500 |
| 26 | RM 205 | 150 | 120, 160 | 100, 200 |
| 27 | RM 447 | 100, 130, 190, 600 | - | - |
| 28 | RM 244 | 180 | 450 | - |
| 29 | RM 8207 | 90, 150 | 110, 160 | 100 |
| 30 | RM 590 | 150, 450 | - | - |
| 31 | RM 206 | 130, 150, 170, 190 | - | - |
| 32 | RM 287 | 90, 100, 110 | - | - |
| 33 | RM 5918 | 200, 270, 450 | 300 | - |
| 34 | RM 3472 | 290, 300, 3100 | 400 | 200, 600 |
| 35 | RM 463 | 200 | 190 | 180 |
| 36 | RM 2529 | 130 | 160, 200,210 | 150, 180 |

Supplementary Table S5: Allelic variation and allele sharing between *O. rufipogon* and *O. nivara* population

| **Population** | **Na** | **NaF** | **Ne** | **LCA** | **He** | **I** |
| --- | --- | --- | --- | --- | --- | --- |
| **24 Parganas(s)** | 2.972 | 2.667 | 2.087 | 0.194 | 0.451 | 0.776 |
| **Angul** | 2.472 | 2.472 | 2.105 | 0.111 | 0.457 | 0.741 |
| **Balangir** | 2.806 | 2.806 | 2.120 | 0.111 | 0.473 | 0.805 |
| **Bankura** | 3.806 | 3.167 | 2.359 | 0.389 | 0.517 | 0.949 |
| **Bauda** | 2.639 | 2.639 | 2.097 | 0.139 | 0.468 | 0.772 |
| **Birbhum** | 3.861 | 3.472 | 2.671 | 0.333 | 0.543 | 1.007 |
| **Bardhaman** | 3.917 | 3.611 | 2.697 | 0.306 | 0.569 | 1.056 |
| **Dhenkanal** | 2.667 | 2.667 | 2.057 | 0.056 | 0.440 | 0.747 |
| **Ganjam** | 3.639 | 3.361 | 2.581 | 0.194 | 0.544 | 0.993 |
| **Jalpaigudi** | 2.194 | 2.194 | 1.933 | 0.056 | 0.417 | 0.651 |
| **Jharsuguda** | 3.111 | 3.111 | 2.188 | 0.167 | 0.463 | 0.829 |
| **Kalahandi** | 3.111 | 3.111 | 2.439 | 0.222 | 0.527 | 0.916 |
| **Keonjhar** | 2.972 | 2.972 | 2.421 | 0.194 | 0.525 | 0.900 |
| **Khurda** | 2.861 | 2.861 | 2.301 | 0.056 | 0.498 | 0.850 |
| **Koraput** | 2.278 | 2.278 | 1.938 | 0.056 | 0.416 | 0.663 |
| **Malda** | 2.778 | 2.778 | 2.267 | 0.111 | 0.474 | 0.811 |
| **Malkangiri** | 2.500 | 2.500 | 2.155 | 0.056 | 0.481 | 0.775 |
| **Mayurbhanj** | 1.889 | 1.889 | 1.729 | 0.028 | 0.349 | 0.529 |
| **Midnapore** | 3.778 | 3.278 | 2.526 | 0.278 | 0.548 | 1.000 |
| **Murshidabad** | 2.806 | 2.806 | 2.274 | 0.139 | 0.490 | 0.834 |
| **North Dinajpur** | 2.639 | 2.639 | 2.091 | 0.194 | 0.455 | 0.760 |
| **Puri** | 2.083 | 2.083 | 1.789 | 0.083 | 0.364 | 0.573 |
| **Purulia** | 2.861 | 2.694 | 2.180 | 0.194 | 0.478 | 0.812 |
| **Rayagada** | 2.139 | 2.139 | 1.875 | 0.056 | 0.398 | 0.621 |
| **South Dinajpur** | 2.667 | 2.667 | 2.169 | 0.167 | 0.474 | 0.791 |
| **Sundargarh** | 3.139 | 2.917 | 2.290 | 0.083 | 0.510 | 0.879 |

Supplementary Table S6: SSR based genetic diversity parameters of wild rice accessions-District wise. Na: average number of allele; NaF: number of allele frequency (≥5%); Ne: average number of effective allele; LCA: least common allele (≤25%); He: Nei’s genetic diversity; I: Shannon’s information index

| **Source** | **df** | **SS** | **MS** | **Est. Var.** | **%** |
| --- | --- | --- | --- | --- | --- |
| **Among Pops** | 25 | 691.877 | 27.675 | 1.026 | 9% |
| **Within Pops** | 414 | 4411.055 | 10.655 | 10.655 | 91% |
| **Total** | 439 | 5102.932 |  | 11.681 | 100% |

Supplementary Table S7: Analysis of molecular variance of wild rice accessions – district wise. df: degree of freedom; SS: Sum of squares; MS: Mean squares; EV: Estimated variance (p<0.001).

| **Sl. No.** | **Accession No.** | **District** | **State** |
| --- | --- | --- | --- |
| *O. nivara* | | | |
| 1 | 100429 | Dakhin Dinajpur | W. Bengal |
| 2 | 100486 | Birbhum | W. Bengal |
| 3 | 100453 | Bardhaman | W. Bengal |
| 4 | 100452 | Bardhaman | W. Bengal |
| 5 | 100431 | Dakhin Dinajpur | W. Bengal |
| 6 | 100451 | Bardhaman | W. Bengal |
| 7 | 100450 | Bardhaman | W. Bengal |
| 8 | 100448 | Bardhaman | W. Bengal |
| 9 | 100432 | Dakhin Dinajpur | W. Bengal |
| 10 | 100426 | 24 Parganas(s) | W. Bengal |
| 11 | 100425 | 24 Parganas(s) | W. Bengal |
| 12 | 100424 | 24 Parganas(s) | W. Bengal |
| 13 | 100423 | 24 Parganas(s) | W. Bengal |
| 14 | 100422 | 24 Parganas(s) | W. Bengal |
| 15 | 100421 | 24 Parganas(s) | W. Bengal |
| 16 | 100420 | 24 Parganas(s) | W. Bengal |
| 17 | 100419 | 24 Parganas(s) | W. Bengal |
| 18 | 100418 | 24 Parganas(s) | W. Bengal |
| 19 | 100417 | 24 Parganas(s) | W. Bengal |
| 20 | 100404 | 24 Parganas(s) | W. Bengal |
| 21 | 100403 | 24 Parganas(s) | W. Bengal |
| 22 | 100428 | Malda | W. Bengal |
| 23 | 100482 | Birbhum | W. Bengal |
| 24 | 100481 | Birbhum | W. Bengal |
| 25 | 100479 | Birbhum | W. Bengal |
| 26 | 100477 | Malda | W. Bengal |
| 27 | 100475 | Murshidabad | W. Bengal |
| 28 | 100471 | Birbhum | W. Bengal |
| 29 | 100468 | Birbhum | W. Bengal |
| 30 | 100467 | Birbhum | W. Bengal |
| 31 | 100463 | Birbhum | W. Bengal |
| 32 | 100460 | Birbhum | W. Bengal |
| 33 | 100297 | Sundargarh | Odisha |
| 34 | 100027 | Kalahandi | Odisha |
| 35 | 100119 | Malkangiri | Odisha |
| 36 | 100338 | Sundargarh | Odisha |
| 37 | 100301 | Balasore | Odisha |
| 38 | 100287 | Sundargarh | Odisha |
| 39 | 100353 | Sambalpur | Odisha |
| 40 | 100360 | Midnapur | W. Bengal |
| 41 | 100228 | Ganjam | Odisha |
| 42 | 100341 | Sundargarh | Odisha |
| 43 | 100316 | Midnapore | W. Bengal |
| 44 | 100288 | Sundargarh | Odisha |
| 45 | 100293 | Sundargarh | Odisha |
| 46 | 100289 | Sundargarh | Odisha |
| 47 | 100008 | Bauda | Odisha |
| 48 | 100328 | Keonjhar | Odisha |
| 49 | 100026 | Kalahandi | Odisha |
| 50 | 100120 | Malkangiri | Odisha |
| 51 | 100043 | Ganjam | Odisha |
| 52 | 100312 | Midnapore | W. Bengal |
| 53 | 100053 | Khurda | Odisha |
| 54 | 100394 | Bankura | W. Bengal |
| 55 | 100010 | Bauda | Odisha |
| 56 | 100347 | Jharsuguda | Odisha |
| 57 | 100016 | Balingir | Odisha |
| 58 | 100214 | Ganjam | Odisha |
| 59 | 100033 | Rayagada | Odisha |
| 60 | 100269 | Dhenkanal | Odisha |
| 61 | 100290 | Sundargarh | Odisha |
| 62 | 100304 | Midnapore | W. Bengal |
| 63 | 100313 | Midnapore | W. Bengal |
| 64 | 100175 | Bardhaman | W. Bengal |
| 65 | 100021 | Kalahandi | Odisha |
| 66 | 100397 | Bankura | W. Bengal |
| 67 | 100023 | Kalahandi | Odisha |
| 68 | 100392 | Bankura | W. Bengal |
| 69 | 100416 | 24 Parganas(s) | W. Bengal |
| 70 | 100388 | Bankura | W. Bengal |
| 71 | 100358 | Midnapur | W. Bengal |
| 72 | 100367 | Bankura | W. Bengal |
| 73 | 100012 | Sonepur | Odisha |
| 74 | 100390 | Bankura | W. Bengal |
| 75 | 100380 | Bankura | W. Bengal |
| 76 | 100384 | Bankura | W. Bengal |
| 77 | 100366 | Bankura | W. Bengal |
| 78 | 100022 | Kalahandi | Odisha |
| 79 | 100052 | Khurda | Odisha |
| 80 | 100011 | Bauda | Odisha |
| 81 | 100476 | Murshidabad | W. Bengal |
| 82 | 100007 | Bauda | Odisha |
| 83 | 100395 | Bankura | W. Bengal |
| 84 | 100385 | Bankura | W. Bengal |
| 85 | 100389 | Bankura | W. Bengal |
| 86 | 100359 | Midnapur | W. Bengal |
| 87 | 100042 | Ganjam | Odisha |
| 88 | 100370 | Bankura | W. Bengal |
| 89 | 100414 | 24 Parganas(s) | W. Bengal |
| 90 | 100386 | Bankura | W. Bengal |
| 91 | 100178 | Balasore | Odisha |
| 92 | 100196 | Sundargarh | Odisha |
| 93 | 100040 | Ganjam | Odisha |
| 94 | 100373 | Bankura | W. Bengal |
| 95 | 100406 | 24 Parganas(s) | W. Bengal |
| 96 | 100185 | Angul | Odisha |
| 97 | 100009 | Bauda | Odisha |
| 98 | 100361 | Midnapur | W. Bengal |
| 99 | 100371 | Bankura | W. Bengal |
| 100 | 100374 | Bankura | W. Bengal |
| 101 | 100399 | Mayurbhanj | Odisha |
| 102 | 100364 | Bankura | W. Bengal |
| 103 | 100095 | Keonjhar | Odisha |
| 104 | 100322 | Keonjhar | Odisha |
| 105 | 100318 | Mayurbhanj | Odisha |
| 106 | 100096 | Koraput | Odisha |
| 107 | 100111 | Malkangiri | Odisha |
| 108 | 100365 | Bankura | W. Bengal |
| *O. rufipogon* | | | |
| 1 | 100430 | Dakhin Dinajpur | W. Bengal |
| 2 | 100488 | Bardhaman | W. Bengal |
| 3 | 100490 | Bardhaman | W. Bengal |
| 4 | 100487 | Birbhum | W. Bengal |
| 5 | 100456 | Bardhaman | W. Bengal |
| 6 | 100455 | Bardhaman | W. Bengal |
| 7 | 100449 | Bardhaman | W. Bengal |
| 8 | 100447 | Hooghly | W. Bengal |
| 9 | 100446 | Malda | W. Bengal |
| 10 | 100445 | Malda | W. Bengal |
| 11 | 100444 | Malda | W. Bengal |
| 12 | 100443 | Malda | W. Bengal |
| 13 | 100442 | Uttar Dinajpur | W. Bengal |
| 14 | 100440 | Uttar Dinajpur | W. Bengal |
| 15 | 100439 | Uttar Dinajpur | W. Bengal |
| 16 | 100438 | Uttar Dinajpur | W. Bengal |
| 17 | 100436 | Jalpaigudi | W. Bengal |
| 18 | 100434 | Uttar Dinajpur | W. Bengal |
| 19 | 100435 | Jalpaigudi | W. Bengal |
| 20 | 100485 | Birbhum | W. Bengal |
| 21 | 100483 | Birbhum | W. Bengal |
| 22 | 100480 | Birbhum | W. Bengal |
| 23 | 100474 | Murshidabad | W. Bengal |
| 24 | 100473 | Murshidabad | W. Bengal |
| 25 | 100472 | Murshidabad | W. Bengal |
| 26 | 100494 | Puri | Odisha |
| 27 | 100470 | Birbhum | W. Bengal |
| 28 | 100409 | 24 Parganas(s) | W. Bengal |
| 29 | 100493 | Puri | Odisha |
| 30 | 100465 | Birbhum | W. Bengal |
| 31 | 100464 | Birbhum | W. Bengal |
| 32 | 100462 | Birbhum | W. Bengal |
| 33 | 100461 | Birbhum | W. Bengal |
| 34 | 100491 | Puri | Odisha |
| 35 | 100458 | Bardhaman | W. Bengal |
| 36 | 100401 | Nadia | W. Bengal |
| 37 | 100354 | Angul | Odisha |
| 38 | 100038 | Ganjam | Odisha |
| 39 | 100495 | - | - |
| 40 | 100035 | Rayagada | Odisha |
| 41 | 100034 | Rayagada | Odisha |
| 42 | 100284 | Sundargarh | Odisha |
| 43 | 100028 | Ganjam | Odisha |
| 44 | 100346 | Jharsuguda | Odisha |
| 45 | 100015 | Balangir | Odisha |
| 46 | 100437 | Jalpaigudi | W. Bengal |
| 47 | 100045 | Ganjam | Odisha |
| 48 | 100019 | Balangir | Odisha |
| 49 | 100457 | Bardhaman | W. Bengal |
| 50 | 100227 | Ganjam | Odisha |
| 51 | 100215 | Ganjam | Odisha |
| 52 | 100046 | Ganjam | Odisha |
| 53 | 100047 | Ganjam | Odisha |
| 54 | 100048 | Ganjam | Odisha |
| 55 | 100049 | Ganjam | Odisha |
| 56 | 100041 | Ganjam | Odisha |
| 57 | 100001 | Dinajpur | W. Bengal |
| 58 | 100013 | Sonepur | Odisha |
| 59 | 100433 | Dakhin Dinajpur | W. Bengal |
| 60 | 100308 | Midnapore | W. Bengal |
| 61 | 100166 | Purulia | W. Bengal |
| 62 | 100141 | Nabarangpur | Odisha |
| 63 | 100030 | Kalahandi | Odisha |
| 64 | 100130 | Koraput | Odisha |
| 65 | 100279 | Jharsuguda | Odisha |
| 66 | 100173 | Bardhaman | W. Bengal |
| 67 | 100262 | Dhenkanal | Odisha |
| 68 | 100170 | Purulia | W. Bengal |
| 69 | 100155 | Bankura | W. Bengal |
| 70 | 100273 | Angul | Odisha |
| 71 | 100323 | Keonjhar | Odisha |
| 72 | 100324 | Keonjhar | Odisha |
| 73 | 100112 | Malkangiri | Odisha |
| 74 | 100161 | Pururia | W. Bengal |
| 75 | 100169 | Purulia | W. Bengal |
| 76 | 100133 | Koraput | Odisha |
| 77 | 100036 | Gajapati | Odisha |
| 78 | 100305 | Midnapore | W. Bengal |
| 79 | 100176 | Bardawan | W. Bengal |
| 80 | 100168 | Purulia | W. Bengal |
| 81 | 100307 | Midnapore | W. Bengal |
| 82 | 100160 | Pururia | W. Bengal |
| 83 | 100314 | Midnapore | W. Bengal |
| 84 | 100315 | Midnapore | W. Bengal |
| 85 | 100167 | Purulia | W. Bengal |
| 86 | 100156 | Bankura | W. Bengal |
| 87 | 100281 | Jharsuguda | Odisha |
| 88 | 100282 | Jharsuguda | Odisha |
| 89 | 100343 | Jharsuguda | Odisha |
| 90 | 100135 | Koraput | Odisha |
| 91 | 100309 | Midnapore | W. Bengal |
| 92 | 100054 | Khurda | Odisha |
| 93 | 100014 | Balangir | Odisha |
| 94 | 100017 | Balangir | Odisha |
| 95 | 100029 | Kalahandi | Odisha |
| 96 | 100006 | Bauda | Odisha |
| 97 | 100334 | Sundargarh | Odisha |
| 98 | 100153 | Bankura | W. Bengal |
| 99 | 100165 | Purulia | W. Bengal |
| 100 | 100152 | Bankura | W. Bengal |
| 101 | 100162 | Purulia | W. Bengal |
| 102 | 100163 | Purulia | W. Bengal |
| 103 | 100002 | Nayagarh | Odisha |
| 104 | 100005 | Khurdha | Odisha |
| 105 | 100004 | Khurdha | Odisha |
| 106 | 100264 | Dhenkanal | Odisha |
| 107 | 100356 | Angul | Odisha |
| 108 | 100174 | Bardhaman | W. Bengal |
| 109 | 100317 | - | - |
| 110 | 100149 | Kalahandi | Odisha |
| 111 | 100320 | Mayurbhanj | Odisha |
| 112 | 100159 | Bankura | W. Bengal |
| 113 | 100310 | Midnapore | W. Bengal |
| 114 | 100265 | Dhenkanal | Odisha |
| 115 | 100266 | Dhenkanal | Odisha |
| 116 | 100351 | Sambalpur | Odisha |
| 117 | 100332 | Sundargarh | Odisha |
| 118 | 100157 | Bankura | W. Bengal |
| 119 | 100154 | Bankura | W. Bengal |
| 120 | 100143 | Nabarangpur | Odisha |
| 121 | 100263 | Dhenkanal | Odisha |
| 122 | 100171 | Bardhaman | W. Bengal |
| 123 | 100350 | Jharsuguda | Odisha |
| 124 | 100018 | Nuapada | Odisha |
| 125 | 100093 | Keonjhar | Odisha |
| 126 | 100164 | Purulia | W. Bengal |
| 127 | 100158 | Bankura | W. Bengal |
| 128 | 100172 | Bardhaman | W. Bengal |
| 129 | 100050 | Ganjam | Odisha |
| 130 | 100270 | - | Odisha |

Supplementary Table S8: *O. nivara* and *O. rufipogon* accessions used in this study

| **SL.NO** | **MARKER** | **SEQUENCE** | **REPEAT MOTIF** | **PRODUCT SIZE** |
| --- | --- | --- | --- | --- |
| 1 | RM 495 | F AATCCAAGGTGCAGAGATGG  R CAACGATGACGAACACAACC | (CTG)7 | 159 |
| 2 | RM10864 | F GAGGTGAGTGAGACTTGACAGTGC  R GCTCATCATCCAACCACAGTCC | GT(27) | 239 |
| 3 | RM3642 | F TCGTTTCCGAGATGTCACTG  R AATTCTCGGGAGAGGGTACG | (GA)14 | 162 |
| 4 | RM6378 | F ATAGGGTGGGTGTGCTGAAC  R AATTCTCGGGAGAGGGTACG | (GAA)19 | 167 |
| 5 | RM423 | F AGCACCCATGCCTTATGTTG  R CCTTTTTCAGTAGCCCTCCC | (TTC)9 | 273 |
| 6 | RM 5780 | F GCTGCTGCATCTTCTACTGC  R ACGCACATGCCTAAGCCTAG | (AGA)14 | 121 |
| 7 | RM422 | F TTCAACCTGCATCCGCTC  R CCATCCAAATCAGCAACAGC | (AG)30 | 385 |
| 8 | RM81B | F GAGTGCTTGTGCAAGATCCA  R CTTCTTCACTCATGCAGTTC | (TCT)10 | 110 |
| 9 | RM3392 | F GTCCAATGATTCGTTCCCAC  R CTTCACCGTTCACCAATTCC | (CT)17 | 164 |
| 10 | RM3866 | F AGTTGGTCATCTACCAGAGC  R GATCTTCTTGCCTCAGAAAG | (GA)29 | 161 |
| 11 | RM261 | F CTACTTCTCCCCTTGTGTCG  R TGTACCATCGCCAAATCTCC | C9(CT)8 | 125 |
| 12 | RM16649 | F CTCCCTTCATGCGTAAGCTCTCC  R GCAAACAGGATCCTCCACAAAGG | (TTA)55 | 341 |
| 13 | RM480 | F GCTCAAGCATTCTGCAGTTG  R GCGCTTCTGCTTATTGGAAG | (AC)30 | 225 |
| 14 | RM413 | F GGCGATTCTTGGATGAAGAG  R TCCCCACCAATCTTGTCTTC | (AG)11 | 79 |
| 15 | RM163 | F ATCCATGTGCGCCTTTATGAGGA  R CGCTACCTCCTTCACTTACTAGT | (GGAGA)4(GA)11C(GA)20 | 124 |
| 16 | RM510 | F AACCGGATTAGTTTCTCGCC  R TGAGGACGACGAGCAGATTC | (GA)15 | 122 |
| 17 | RM204 | F GTGACTGACTTGGTCATAGGG  R GCTAGCCATGCTCTCGTACC | (CT)44 | 169 |
| 18 | RM528 | F GGCATCCAATTTTACCCCTC  R AAATGGAGCATGGAGGTCAC | (AGAT)9 | 232 |
| 19 | RM336 | F CTTACAGAGAAACGGCATCG  R GCTGGTTTGTTTCAGGTTCG | (CTT)18 | 154 |
| 20 | RM253 | F TCCTTCAAGAGTGCAAAACC  R GCATTGTCATGTCGAAGCC | (GA)25 | 141 |
| 21 | RM3404 | F AGTCCTGAGTCTCCTGTCCT  R CCTGTTCGATCTTGAACTTC | (CT)17 | 136 |
| 22 | RM8020 | F ATCCTCGATGAATTGTATAT  R GAAGAGGTGTACATGAATAA | (TA)20(GA)19 | 167 |
| 23 | RM72 | F CCGGCGATAAAACAATGAG  R GCATCGGTCCTAACTAAGGG | (TAT)5C(ATT)15 | 166 |
| 24 | RM547 | F TAGGTTGGCAGACCTTTTCG  R GTCAAGATCATCCTCGTAGCG | (ATT)19 | 235 |
| 25 | RM215 | F CAAAATGGAGCAGCAAGAGC  R TGAGCACCTCCTTCTCTGTAG | (CT)16 | 148 |
| 26 | RM205 | F CTGGTTCTGTATGGGAGCAG  R CTGGCCCTTCACGTTTCAGTG | (CT)25 | 122 |
| 27 | RM447 | F CCCTTGTGCTGTCTCCTCTC  R ACGGGCTTCTTCTCCTTCTC | (CTT)8 | 111 |
| 28 | RM244 | F CCGACTGTTCGTCCTTATCA  R CTGCTCTCGGGTGAACGT | (CT)4(CG)3C(CT)6 | 163 |
| 28 | RM8207 | F TTCATCGACATCATCAACTG  R CAGTTTGGGATGAAGTGTTC | (TTC)23 | 191 |
| 30 | RM590 | F CATCTCCGCTCTCCATGC  R GGAGTTGGGGTCTTGTTCG | (TCT)10 | 137 |
| 31 | RM206 | F CCCATGCGTTTAACTATTCT  R CGTTCCATCGATCCGTATGG | (CT)21 | 147 |
| 32 | RM287 | F TTCCCTGTTAAGAGAGAAATC  R GTGTATTTGGTGAAAGCAAC | (GA)21 | 118 |
| 33 | RM5918 | F ATGGCTGTCGATTGGCTATC  R CTGCTAGTGCTTGCAACCAG | (ATT)24 | 201 |
| 34 | RM3472 | F ATCGCAAGAACTCCGTGAAG  R CGCTTTTGAGCTCGCCTC | (CT)21 | 215 |
| 35 | RM463 | F TTCCCCTCCTTTTATGGTGC  R TGTTCTCCTCAGTCACTGCG | (TTAT)5 | 192 |
| 36 | RM2529 | F CATTAAAATCAGTGGGACTG  R AGGCATTTCCTGATATGATC | (AT)29 | 134 |

Supplementary Table S9: Details of hvSSRs used in the study
